# Survival of livestock-associated methicillin-resistant *Staphylococcus aureus* CC398 on different surface materials

**DOI:** 10.1186/s13028-023-00676-z

**Published:** 2023-03-21

**Authors:** Krista Tuominen, Sara Frosth, Karl Pedersen, Thomas Rosendal, Susanna Sternberg Lewerin

**Affiliations:** 1 Department of Biomedical Sciences and Veterinary Public Health, Swedish University of Agricultural Sciences, Box 7036, SE‐750 07 Uppsala, Sweden; 2 Department of Animal Health and Antimicrobial Strategies, National Veterinary Institute, SE‐751 89 Uppsala, Sweden; 3 Department of Disease Control and Epidemiology, National Veterinary Institute, SE‐751 89 Uppsala, Sweden

**Keywords:** Bacterial decay, Concrete, Environment, Half-life, LA-MRSA, Plastic, Steel

## Abstract

**Background:**

Zoonotic livestock-associated methicillin-resistant *Staphylococcus aureus* (LA-MRSA) is widely spread in pig herds in many countries. However, the knowledge regarding the survival of LA-MRSA in the pig farm environment is currently limited. The aim of this study was to assess the survival of LA-MRSA on different surface materials found in the farm environment. The study investigated the survival of two different LA-MRSA strains belonging to the clonal complex (CC) 398 on four different surfaces: stainless steel, polypropylene plastic, K30 concrete and commercial concrete disk coupons. The survival of the bacteria over time was determined by the viable count method and, where possible, fitting a model to the observed data by using nonlinear least squares method to calculate the half-life (${t}_{1/2}$) for different strain and material combinations.

**Results:**

The study showed that the half-life of the bacteria was longer on polypropylene plastic (${t}_{1/2}$=11.08–15.78 days) than on stainless steel (${t}_{1/2}$=2.45–7.83 days). On these materials, both LA-MRSA strains survived through the 14 week observation period. The bacterial decay was fastest on the concrete surfaces, where LA-MRSA became undetectable after 3–9 weeks.

**Conclusions:**

The survival of LA-MRSA in the pig farm environment may be affected by different surface materials. A more frequent sampling protocol (< 7 days) is needed to determine the half-life on concrete surfaces.

## Background

Livestock-associated methicillin-resistant *Staphylococcus aureus* (LA-MRSA) belonging to clonal complex (CC) 398 is a zoonotic pathogen that colonizes several animal species with pigs being one of its main reservoirs [1, 2]. Even though LA-MRSA rarely causes clinical infections in pigs, it poses a public health risk especially to those working with livestock [3, 4]. However, the spread of LA-MRSA is not limited to direct contact between humans and pigs as spillover to persons without livestock contact has also been reported [5–7]. While the relative contribution of indirect transmission within a pig herd is uncertain, previous studies have proposed that transmission through the environment plays a part in the spread of LA-MRSA [8–10]. Bioaerosols have been proposed as one possible route of environmental transmission of LA-MRSA [9] as well as the contamination of the environment, feed and material [11]. Additionally, humans working at or visiting pig farms are possible sources of LA-MRSA introduction to the herd [12]. A study by Feld et al. [13] assessed the survival of LA-MRSA in the dust of pig farms, but the survival on different surface materials commonly found in a pig farm is largely unknown.

The survival of bacteria is dependent on nutrients and external factors such as temperature, humidity, pH and oxygen concentration, but the optimal environment varies between different bacteria. The temperature and pH range for the growth of *S. aureus* in general is 7–48 °C and 4.0–10.0, respectively [14]. According to previous studies, *S. aureus* survives better in lower relative humidity (34%) and the survival declines as the relative humidity increases [15, 16]. *Staphylococcus aureus* is also capable of forming biofilms, which improves its survival in challenging conditions [17, 18].

The survival can vary between different *S. aureus* strains, which suggests that the intrinsic factors of the bacteria are also important. In a study of nosocomial MRSA by Wagenvoort et al. [19], outbreak isolates were found to survive longer than isolates from sporadic cases, where the outbreak isolates were reported to survive up to 6–9 months in *in-vitro* environment. In another study, a nosocomial MRSA strain was found to survive longer than a methicillin-susceptible *S. aureus* (MSSA) strain and the presence of dust of hospital origin was found to increase the survival times for both MRSA and MSSA [20]. The effect of surface materials on the survival of *S. aureus* has been previously studied in a hospital setting, where *S. aureus* remained viable for at least 1 week on all tested hospital surface materials [21]. However, whether or not the survival of LA-MRSA differs from the hospital-associated MSSA and MRSA strains is largely unknown.

This study focused on investigating the survival time of LA-MRSA strains belonging to CC398 on different surface materials (polypropylene plastic, stainless steel and concrete) that are commonly found in indoor pig facilities. Plastic, such as polypropylene, is used in surfaces such as slatted floors, pen dividers and equipment, while stainless steel is used in crate structures as well as in feeders and water nipples. Concrete is widely used as a flooring material in pig farms and can be considered as a harsh material to bacteria due its alkaline, dry and salty properties [22].

The aim of the study was to provide input parameters for an ongoing modelling study and to support decision making regarding the control and sanitation practices against LA-MRSA. To achieve this, the survival of LA-MRSA CC398 was measured on different materials commonly used in the pig farm environment.

## Methods

### Surface materials

Four different surface materials were used in the study: stainless steel, polypropylene plastic (PP) and two types of concrete. The stainless steel was in the form of sterile 15 mm diameter custom made disks (EN 1.4301; DHinox AB, Uppsala, Sweden) and the polypropylene plastic was screw caps from sterile 15 ml centrifuge tubes (Sarstedt, Nümbrecht, Germany). The two types of concrete were 12.7 mm diameter concrete coated polycarbonate disc coupons (BioSurface Technologies, Bozeman, Montana, USA) and large concrete cylinders. The concrete in the disk coupons was type ½ Portland cement with 200-micron foundry sand as aggregate material. The concrete cylinders were type K30, which is the most common type of concrete used in Swedish pig farms (personal communication, president of Swedish Pig Farmers’ Association). The cylinders were broken into pieces of approximately 5–7 cm in diameter. All materials were autoclaved prior to inoculation.

### Bacterial strains

Two different *S. aureus* CC398 strains were used to contaminate the surface materials. Both strains (S1 and S2) originated from two Danish field studies: the S1 strain was obtained from the SPACE project [23] and the S2 strain from the BioVir project [24]. The identifiers for the strains were ”SPACE sek. 1 gr. 3 hold 1 3/10-19 B10 MRSA 16/10-19” and “BioVir 5b/S3/control W52 sow 6 pig 2”, respectively. Both strains were LA-MRSA CC398 and belonged to spa-type t034.

The strains had been stored at − 70 °C in Brain Heart Infusion (BHI) broth (CM1135; Thermo Fisher Scientific Inc., Waltham, MA, USA) with 15% glycerol, before they were subcultured twice on 5% bovine blood agar (B341960; National Veterinary Institute, Uppsala, Sweden) and on selective Oxoid Brilliance MRSA 2 agar (PO5310A, Thermo Fisher Scientific Inc.).

### Bacterial counts

The plates were incubated at 37 °C for 24 h. One colony of each subcultured strain was inoculated into 50 mL of BHI broth (Thermo Fisher Scientific Inc.) and incubated at 37 °C for 24 h. A viable count was performed by plating 100 μL of each broth culture as ten-fold serial dilutions on the selective agar as well as on the 5% bovine blood agar to compare the results between different media. The plates were incubated at 37 °C for 48 h (read after 24 h and 48 h).

From each broth culture, 100 µL was applied to the different surface materials. The bacterial concentrations of the broths are presented in Table 1. The contaminated steel, plastic and concrete disk coupons were placed on petri dishes (92 × 16 mm; Sarstedt). The large concrete pieces were stored in 1000 mL polypropylene sample containers (VWR International, Leuven, Belgium). All samples were air-dried for 4 h and then stored on a laboratory bench (room temperature 20–22 °C, relative humidity 66–68%) throughout the experiment, covered with the petri dish lids.

**Table 1:** The estimated concentration of LA-MRSA in broth and in the initial material samples

Material	Strain	Plate^a^	Broth (CFU/mL)	Sample (CFU/mL)
Steel	S1	BA	8.80 × 10^9^	6.60 × 10^9^
		MRSA	7.20 × 10^9^	1.34 × 10^9^
	S2	BA	1.33 × 10^10^	8.23 × 10^9^
		MRSA	5.50 × 10^9^	1.48 × 10^9^
Plastic	S1	BA	8.80 × 10^9^	1.33 × 10^10^
		MRSA	7.20 × 10^9^	1.47 × 10^9^
	S2	BA	1.33 × 10^10^	7.43 × 10^9^
		MRSA	5.50 × 10^9^	8.57 × 10^8^
Concrete disk	S1	BA	1.59 × 10^10^	3.60 × 10^9^
		MRSA	7.30 × 10^9^	1.86 × 10^9^
	S2	BA	1.48 × 10^10^	4.40 × 10^9^
		MRSA	1.03 × 10^10^	1.53 × 10^9^
Concrete piece	S1	BA	1.06 × 10^10^	2.16 × 10^9^
		MRSA	7.70 × 10^9^	2.18 × 10^8^
	S2	BA	1.17 × 10^10^	5.90 × 10^8^
		MRSA	6.60 × 10^9^	2.26 × 10^8^

The livestock-associated methicillin-resistant *Staphylococcus aureus* (LA-MRSA) concentrations for two strains (S1 and S2) were estimated in broth and in the initial material samples (steel, plastic, concrete disk and concrete disk). The estimated broth concentrations were from single value and the estimated sample concentrations were mean concentrations from triplicate samples. The concentrations were calculated as viable counts on both 5% bovine blood agar and selective Oxoid Brilliance MRSA 2 agar

^a^BA = 5% bovine blood agar, MRSA = selective Oxoid Brilliance MRSA 2 agar

An initial viable count for each material was performed after the samples had dried. This was done by placing one material sample into a 50 mL centrifugation tube (Sarstedt) with 5 g of 3 mm glass beads (soda-lime glass; Merck KGaA, Darmstadt, Germany) and 10 mL of buffered peptone water (1% peptone, 8.5% NaCl), followed by shaking for 3 min in 660 rpm in an orbital shaker (Yellow line OS 2 basic; IKA-Werke GmbH & Co. KG, Staufen, Germany). The large pieces of concrete were immersed in 100 mL of peptone water with 50 g of glass beads. From the suspension, ten-fold serial dilution was prepared using a Dilushaker III (LabRobot, Stenungsund, Sweden). From the serial dilution, 100 μL was plated on selective MRSA plates and 5% bovine blood agar plates and incubated at 37 °C for 48 h (read after 24 h and 48 h). For the following viable counts, all sample triplicates were plated on selective MRSA plates and one triplicate from each sample was also plated on 5% bovine blood agar. Using selective media was seen as suitable for this study as it reduces the risk of contamination of the plates by other bacteria. The viable counts for plastic and steel were continued at weekly intervals for a total of 14 weeks. For the concrete samples, the first two viable counts were performed at 1 week intervals and the subsequent counts once every 2 weeks for a total period of 5 and 11 weeks for the concrete disks and the large concrete pieces, respectively.

### Confirmation of the strains

Suspected MRSA colonies from the beginning and the end of the study for each material and strain combination were confirmed to be MRSA by using a qPCR assay detecting *mecA*, *mecC*, *nuc* and PVL genes as previously described by Pichon et al. [25]. The qPCR was run with the following modifications: two duplex assays were run instead of one quadruplex assay (*nuc*/PVL and *mecA*/*mecC*, respectively) and the TaqMan Fast Advanced Master Mix (Thermo Fisher Scientific Inc.) was used instead of the QuantiFast Multiplex PCR kit from Qiagen. The assays were run on the CFX Opus 96 Real-Time PCR Instrument (Bio-Rad Laboratories Inc., Hercules, CA USA) and analysed by the CFX Maestro Software version 2.0 (Bio-Rad Laboratories Inc.) with default settings. The strains CCUG 60578 and CCUG 63582 (Culture Collection, University of Gothenburg, Sweden) were used as positive controls and DNase- and RNase free water (W4502; Merck KGaA) as negative control. Prior to the qPCR, the strains were subcultured on blood agar and incubated at 37 °C for 24 h. Approximately 3 μL of each culture was suspended to 180 μL of lysis buffer G2 (Qiagen, Hilden, Germany) in a 2 mL microcentrifuge tube. Ten μL of lysozyme (100 mg/mL; Merck KGaA) and 10 µL lysostaphin (5 mg/mL; Merck KGaA) was added to the microcentrifuge tubes and vortexed prior to incubation in a ThermoMixer C (Eppendorf, Hamburg, Germany) at 37 °C and 300 rpm for 90 min. The DNA was extracted with EZ1 Advanced XL (Qiagen) and EZ1 DNA tissue kit (Lot. No. 169044160) using the bacterial protocol and 100 μL elution volume.

### Bacterial decay models

The observed bacterial counts on the selective MRSA plates were analysed by using nonlinear least squares regression (NLS). This was performed by using the nls-function (Nonlinear Least Squares) in R version 4.2.0 [26]. The bacterial decay was described with an exponential model $$N(t)={N}_{0}{e}^{-\lambda t}$$, which can be linearized to $$\mathrm{ln}N(t)=\mathrm{ln} {N}_{0}- \lambda t+{\varepsilon }_{t}$$, where N(t) represents the CFU number (CFU/mL) of bacteria at time $$t$$, $${N}_{0}$$ is the CFU number of bacteria at $$t=0$$, λ is the rate of decay and $${\varepsilon }_{t}$$ represents the error term. The half-life ($$t_{{{\raise0.5ex\hbox{$\scriptstyle 1$} \kern-0.1em/\kern-0.15em \lower0.25ex\hbox{$\scriptstyle 2$}}}}$$) was obtained from the decay rates by $$t_{{{\raise0.5ex\hbox{$\scriptstyle 1$} \kern-0.1em/\kern-0.15em \lower0.25ex\hbox{$\scriptstyle 2$}}}} = \ln \left( 2 \right)/ \lambda$$. The model was fitted separately for the different bacterial strain and surface material combinations.

## Results

### Initial bacterial concentrations

The LA-MRSA concentrations in broth and the initial mean concentrations recovered from samples are presented in Table 1. In most cases, the mean bacterial concentrations obtained from the sample materials were lower than the concentrations of the corresponding broth that was used for preparing the samples. The initial mean bacterial concentrations on selective LA-MRSA plates were consistently lower than the corresponding samples on 5% bovine blood agar plates.

### Bacterial counts on plastic and steel over time

**Fig. 1:**
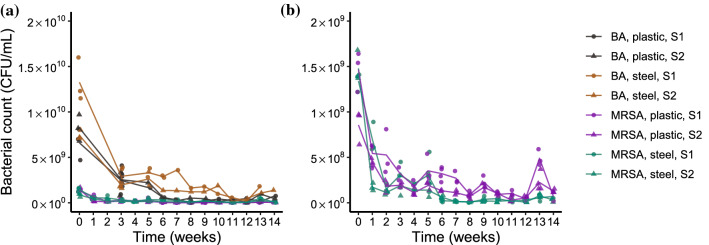
The livestock-associated methicillin-resistant *Staphylococcus aureus* counts (CFU/mL) on steel and plastic surfaces over time. The points represent the viable counts within the quantification range (30–300 CFU) that were obtained from the samples; the lines represent the mean viable count of each material, strain and plate combination. **a** 5% bovine blood agar (BA) and selective Oxoid Brilliance MRSA 2 agar (MRSA). **b** selective MRSA plates from the same data. The counts from strain S1 steel sample on blood agar at week 14 are missing due to an error in sample preparation

The viable cell counts on plastic and steel samples are presented in Fig. 1. The observed bacterial counts followed an expected exponential decay up to week 14 when an increase was observed on both steel and plastic samples. During the observation period, complete die out of the bacteria was not observed for either strain on these surface materials. The blood agar plates yielded larger number of colonies than the corresponding selective MRSA plates that were prepared from the same sample. Mixed culture growth (suspected contamination, i.e., not *S. aureus*) was observed in some of the blood agar plates, which were excluded from the results.

### Bacterial counts on concrete over time

**Fig. 2:**
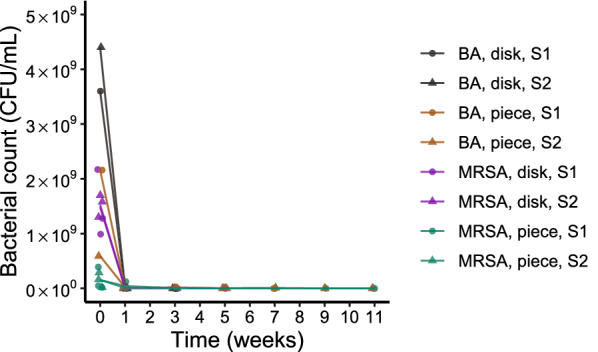
The livestock-associated methicillin-resistant *Staphylococcus aureus* counts (CFU/mL) on concrete surfaces over time. Two different concrete surfaces were used (concrete disks and concrete pieces). Concrete disks were observed for 4 weeks and concrete pieces for 11 weeks. All strain and material combinations were plated in triplicates on Oxoid Brilliance MRSA 2 agar (MRSA) and as a single sample on 5% bovine blood agar (BA). The points represent the observed viable counts; counts with less than 30 CFU are also included in the figure. If a sample had a count below the quantification limit (< 30) on multiple dilution plates, the count from lowest dilution is presented. The lines represent the mean viable count of each concrete type, strain and plate combination

The bacterial counts on concrete disks and concrete pieces are presented in Fig. 2. The observed counts of both bacterial strains decreased very rapidly on both concrete surfaces. Due to the rapid decrease, the time interval for sampling was unable to capture the exponential nature of bacterial decay even when the bacterial counts below quantification limit (< 30 CFU) were included in the results. The blood agar plates yielded larger numbers of colonies than the corresponding selective MRSA plates that were prepared from the same sample. Mixed culture growth (suspected contamination) was observed on some of the blood agar plates, which were excluded from the results.

LA-MRSA became undetectable on all concrete material and strain combinations during the observation period. The observed die out for each combination is summarized in Table 2.

**Table 2:** Time until LA-MRSA became undetectable (die out) on viable count plates prepared from concrete samples

Material	Strain	Plate^**a**^	Time to die out (weeks)
Concrete disk	S1	BA	5
Concrete disk	S1	MRSA	5
Concrete disk	S2	BA	3
Concrete disk	S2	MRSA	3
Concrete piece	S1	BA	5
Concrete piece	S1	MRSA	7
Concrete piece	S2	BA	9
Concrete piece	S2	MRSA	9

Two different types of concrete (concrete disk and concrete piece) were used as sample materials. The livestock-associated methicillin-resistant *Staphylococcus aureus* (LA-MRSA) were determined to have died out when the bacteria had become undetectable on all viable count plates. The viable counts were prepared on 5% bovine blood agar and selective Oxoid Brilliance MRSA 2 agar. The first two viable counts were performed at 1 week intervals and the subsequent counts at biweekly intervals for a total period of 5 weeks for the concrete disks and 11 weeks for the large concrete pieces

^a^BA = 5% bovine blood agar, MRSA = selective Oxoid Brilliance MRSA 2 agar

### Confirmation of the strains

The suspected LA-MRSA colonies recovered from the prepared samples were confirmed to be LA-MRSA by using qPCR. All of the material and strain combinations were determined to be *mecA* and *nuc* positive and *mecC* and PVL negative.

### Bacterial decay models

The estimated decay constants and half-lives for steel and plastic surfaces are presented in Table 3. When the exponential decay models were fitted to the bacterial count data for these surface materials, the residuals of the fitted models for each material and strain combination were not normally distributed for any of the strain and material combinations. The fitted intercepts and coefficients were significantly different from 0 (P ≤ 0.05). On concrete, the quick die out of the bacteria provided only a limited number of points to fit the NLS model and the data did not have equal variance over all ranges of the explanatory variable (time). Therefore, it was not possible to fit a model to this dataset.

**Table 3:** The decay of livestock-associated methicillin-resistant *Staphylococcus aureus* on steel and plastic surfaces

Material	Strain	Decay constant (λ)/day^a^	SE (λ)^b^	Half-life (${t}_{1/2}$) in days^a^
Steel	S1	0.089 (0.070–0.107)	0.009	7.83 (6.45–9.96)
	S2	0.283 (0.212–0.354)	0.035	2.45 (1.96–3.27)
Plastic	S1	0.044 (0.031–0.057)	0.006	15.78 (12.22–22.26)
	S2	0.063 (0.045–0.080)	0.009	11.08 (8.70–15.28)

The presented values were obtained by fitting a model to observed bacterial data by using nonlinear least squares (NLS) method. Separate models were fitted to two bacterial strains (S1 and S2) on two surface materials (steel and plastic)

^a^Corresponding 95% confidence intervals are presented in parenthesis

^b^Standard error for daily decay constant (λ)

## Discussion

This study investigated the survival of LA-MRSA CC398 on different materials commonly used in the pig farm environment. Surface materials that allow the bacteria to persist in the environment will contribute to the risk of LA-MRSA carriers, not only among the pigs but also among humans working in the farm environment. Identifying such risk surface materials is beneficial for planning effective cleaning and disinfection protocols in farms. While cleaning and disinfection have been shown to be efficient in reducing LA-MRSA, routine cleaning procedures are not sufficient to remove all LA-MRSA from the environment [27]. However, cleaning and disinfection after culling the entire herd has been proven to be efficient in eradicating LA-MRSA from a pig farm [28].

The results of this study suggest that the survival time of LA-MRSA CC398 varies depending on the surface material. Of the materials used in the study, concrete seems to be the most unfavourable surface for LA-MRSA. Even though estimating the half-life using a modelling approach was not possible for the concrete data, this conclusion is supported by the observation that the used LA-MRSA strains became undetectable on the concrete surfaces in 3–9 weeks, while on polypropylene plastic and stainless steel the bacteria were still viable after 14 weeks. However, the bacterial counts on the concrete should be assessed with caution as there remains uncertainty in the proportion of the bacteria that was detectable from the concrete surfaces. As concrete is capable of absorbing water [29], some of the bacterial suspension could have been absorbed and trapped in the concrete samples. Therefore, the bacteria inside the bigger concrete pieces may not have been detected with the methods used in this study. However, LA-MRSA also diminished quickly from the thin concrete disks, which, together with the radical drop of weekly bacterial counts between the first two observation time points, suggests that concrete is a hostile surface for LA-MRSA. This observation is further supported by a study by Maresca et al. [22] where the microflora on concrete was shown to have relatively low bacterial diversity and most of the identified bacteria belonged to the Actinobacteria and Proteobacteria phyla.

Many textbooks and standards recommend counting only culture plates with colony numbers between 30 and 300 [30, 31]. However, in this study, the viable counts on concrete that were below these quantification limits were included in the results to illustrate the rapid decrease in the bacterial counts between the first and second observation time points. Due to the uncertainty and the low number of quantifiable observations for concrete, the presented bacterial count results need to be interpreted with caution. Further studies with a shorter sampling interval are needed to be able to fit a model and calculate a half-life for the survival of LA-MRSA on concrete.

In this study, the non-linear least squares method was used to fit models to the polypropylene plastic and stainless steel data. The obtained fits for different material and strain combinations were imperfect, but this approach was considered to best represent the bacterial decay which is assumed to be exponential in the absence of active growth. Fitting an NLS-model to the data generated an average decay, but was unable to capture the increase in bacterial counts which was observed at the end of the study for the steel and plastic samples.

Based on results presented in Table 3, LA-MRSA survives longer on polypropylene plastic surface than on stainless steel or concrete. The mean half-life for plastic (*t*_½_ = 15.8 and 11.1 days for strains S1 and S2, respectively) was also longer than what has been previously reported in dust (*t*_½_ = 5 days) in the study by Feld et al. [13]. These results indicate that plastic surfaces could allow LA-MRSA CC398 to persist in the environment longer than on the other studied materials or in dust. However, in a pig farm environment the different surfaces can be assumed to be at least partially covered by organic material and therefore the survival of LA-MRSA in the pig farm environment is likely to be an interplay between several factors. These factors also include the environmental conditions (e.g., temperature and humidity) and the intrinsic properties of different bacterial strains. Additionally, surfaces in the barn environment deteriorate over time, making them rough and potentially more favourable for bacterial survival than what was observed in this *in-vitro* study. Nevertheless, these results suggest that considering the properties of the surface materials may improve the results of on-farm cleaning and disinfection routines. From a disease modelling perspective, more data about the survival and decay of LA-MRSA can support accurate model development.

The strains obtained for this study were field samples from Danish studies. As both of the strains belonged to the same spa-type, they do not represent the whole spectrum of different LA-MRSA CC398 strains. Using these particular isolates was seen as justified since they represent LA-MRSA, which have been encountered in a pig farm environment and were recovered from well-known farms with high traceability. The fitted decay constants and the corresponding calculated half-lives indicate that there is a difference in survival between different LA-MRSA CC398 strains. This is an interesting result as one could assume that LA-MRSA strains belonging to the same spa-type would behave similarly. Further research including more diverse strains of LA-MRSA would be required to understand the variation of survival times. In this study the LA-MRSA strain S2 appeared to have a shorter half-life on plastic and steel than strain S1. The variation in survivability of different strains was more pronounced on concrete and steel than on plastic. Interestingly, on concrete the S1 strain stayed detectable longer than the S2 strain, but due to the uncertainties related to the concrete data, interpretations of the concrete results should be made with caution.

The viable count method was used to estimate bacterial concentrations and each strain and material combination was counted in triplicate on selective MRSA agar. However, the selective MRSA plates are commonly used for the detection of the bacteria rather than for quantification purposes, and it is likely to be a more demanding medium for the bacteria to grow on. The purpose of selective media is to inhibit the growth of non-target bacteria, while they may not provide the optimal growth conditions for the target bacteria. This phenomenon is also known for other types of selective media, which means that counts on selective media are underestimating the true number of bacteria. To monitor the possible difference between selective and non-selective media, one of each sample triplicate was also plated on 5% bovine blood agar. The results shown in Fig. 1, Fig. 2 and Table 1 all demonstrate that the viable counts on selective MRSA agar were systematically lower than the counts on the corresponding blood agar plates. However, based on visual observation of the data, the relationship between the different media remained sufficiently stable over time. Therefore, using the selective MRSA agar was seen as justified since the decay is likely to be similar on both media and since it affected all samples equally.

During the study period, fluctuations in the bacterial counts on the plastic and steel surfaces were observed. Generally, this could be seen as normal variation, but on week 14 all the bacterial counts increased simultaneously. Different technical errors in the laboratory were considered, but no clear reason was identified to explain this increase. This raised questions regarding the possible dynamics of the decay of LA-MRSA; perhaps biological processes such as biofilm formation could have affected the observed viable counts at this time point. The reason for the increased counts remains open and additional studies would be needed to identify whether this phenomenon recurs.

This study did not look into the differences in biofilm formations on different surface materials, but bacterial biofilms are known to be more resilient to environmental challenges than planktonic cells [32]. According to a study by Lee et al. [33], *S. aureus* had a higher level of biofilm formation on hydrophilic surfaces (e.g., stainless steel) than on hydrophobic surfaces (e.g., polypropylene plastic), but the formation increased on hydrophobic surfaces when 1% glucose was supplemented to the growth media. The authors also concluded that the ability to form biofilms varied between the different *S. aureus* strains. This contrast between hydrophilic and hydrophobic surfaces was not observed in the current study, but the results are not directly comparable. It remains uncertain what kind of combined effect the different surface material properties, the LA-MRSA CC398 strains and the BHI broth had on the survival of the bacteria or how much biofilm was formed on the different materials. As the BHI broth was not washed from the bacteria before contaminating the materials, the nutrients of the broth would likely have supported the survival of the bacteria. However, conserving the BHI broth when contaminating the samples was seen as justified, as it was regarded to be more analogous to conditions where bacteria are shed to the environment together with organic material, such as skin cells or secretions, than it would have been if the suspension had not had any nutrients.

## Conclusions

The survival of LA-MRSA in the pig farm environment may be different on different surface materials in the farm. LA-MRSA survives longer on polypropylene plastic than on stainless steel or concrete mixture. Concrete seems to be an unfavourable material for LA-MRSA but further research is needed to determine half-life on concrete surfaces. The findings highlight the importance of bacterial contamination of the environment for on-farm persistence of LA-MRSA and the relevance of effective cleaning and disinfection routines for mitigating spread. Additionally, the data presented can help to improve the input parameters of LA-MRSA models.

## Data Availability

The datasets used and/or analysed during the current study are available from the corresponding author on reasonable request.

## References

[1] Graveland H, Duim B, van Duijkeren E, Heederik D, Wagenaar JA (2011). Livestock-associated methicillin-resistant *Staphylococcus aureus* in animals and humans. Int J Med Microbiol.

[2] Verkade E, Kluytmans J (2014). Livestock-associated *Staphylococcus aureus* CC398: animal reservoirs and human infections. Infect Genet Evol.

[3] Goerge T, Lorenz MB, van Alen S, Hubner NO, Becker K, Kock R (2017). MRSA colonization and infection among persons with occupational livestock exposure in Europe: prevalence, preventive options and evidence. Vet Microbiol.

[4] Chen C, Wu F (2021). Livestock-associated methicillin-resistant *Staphylococcus aureus* (LA-MRSA) colonisation and infection among livestock workers and veterinarians: a systematic review and meta-analysis. J Occup Environ Med.

[5] Deiters C, Gunnewig V, Friedrich AW, Mellmann A, Kock R (2015). Are cases of methicillin-resistant *Staphylococcus aureus* clonal complex (CC) 398 among humans still livestock-associated?. Int J Med Microbiol.

[6] Larsen J, Petersen A, Sorum M, Stegger M, Lv Alphen, Valentiner-Branth P (2015). Meticillin-resistant *Staphylococcus aureus* CC398 is an increasing cause of disease in people with no livestock contact in Denmark, 1999 to 2011. Eurosurveillance.

[7] The Danish Integrated Antimicrobial Resistance Monitoring and Research Programme. DANMAP 2020—Use of antimicrobial agents and occurrence of antimicrobial resistance in bacteria from food animals, food and humans in Denmark. 2020. Accessed 16 Mar 2023.

[8] Friese A, Schulz J, Hoehle L, Fetsch A, Tenhagen BA, Hartung J (2012). Occurrence of MRSA in air and housing environment of pig barns. Vet Microbiol.

[9] Bos ME, Verstappen KM, van Cleef BA, Dohmen W, Dorado-Garcia A, Graveland H (2016). Transmission through air as a possible route of exposure for MRSA. J Expo Sci Environ Epidemiol.

[10] Rosen K, Roesler U, Merle R, Friese A (2018). Persistent and transient airborne MRSA colonization of piglets in a newly established animal model. Front Microbiol.

[11] Crombe F, Argudin MA, Vanderhaeghen W, Hermans K, Haesebrouck F, Butaye P (2013). Transmission dynamics of methicillin-resistant *Staphylococcus aureus* in pigs. Front Microbiol.

[12] Grøntvedt CA, Elstrøm P, Stegger M, Skov RL, Skytt Andersen P, Larssen KW (2016). Methicillin-resistant *Staphylococcus aureus* CC398 in humans and pigs in norway: a “one health” perspective on introduction and transmission. Clin Infect Dis.

[13] Feld L, Bay H, Angen O, Larsen AR, Madsen AM (2018). Survival of LA-MRSA in dust from swine farms. Ann Work Expo Health.

[14] International Commission on Microbiological Specifications for Foods (1996). Microorganisms in foods 5: microbiological specifications of food pathogens.

[15] Lidwell OM, Lowbury EJ (1950). The survival of bacteria in dust II the effect of atmospheric humidity on the survival of bacteria in dust. J Hyg.

[16] McEldowney S, Fletcher M (1988). The effect of temperature and relative humidity on the survival of bacteria attached to dry solid surfaces. Lett Appl Microbiol.

[17] Flemming H-C, Wingender J, Szewzyk U, Steinberg P, Rice SA, Kjelleberg S (2016). Biofilms: an emergent form of bacterial life. Nat Rev Microbiol.

[18] Shen J, Wang H, Zhu C, Zhang M, Shang F, Xue T (2021). Effect of biofilm on the survival of *Staphylococcus aureus* isolated from raw milk in high temperature and drying environment. Food Res Int.

[19] Wagenvoort JHT, Sluijsmans W, Penders RJR (2000). Better environmental survival of outbreak vs. sporadic MRSA isolates. J Hosp Infect.

[20] Wagenvoort JHT, Penders RJR (1997). Long-term in-vitro survival of an epidemic MRSA phage-group III-29 strain. J Hosp Infect.

[21] Katzenberger RH, Rösel A, Vonberg R-P (2021). Bacterial survival on inanimate surfaces: a field study. BMC Res Notes.

[22] Maresca JA, Moser P, Schumacher T (2017). Analysis of bacterial communities in and on concrete. Mater Struct.

[23] Ministry of Food, Agriculture and Fisheries of Denmark. Projektet SPACE—Renovering og udvidelse af en eksisterende stald med 3870 stipladser ved Randers (in Danish). 2022. https://gudp.lbst.dk/projekter/gudp-projekter/projektet-space-renovering-og-udvidelse-af-en-eksisterende-stald-med-3870-stipladser-ved-randers/. Accessed 16 Mar 2023.

[24] Bækbo P, Sommer HM, Espinosa-Gongora C, Pedersen K, Toft N. Ingen effekt af biocidet BioVir på forekomst af MRSA i stalden (in Danish). Seges Danish Pig Research Centre. 2018. https://svineproduktion.dk/publikationer/kilder/lu_medd/2018/1144. Accessed 16 Mar 2023.

[25] Pichon B, Hill R, Laurent F, Larsen AR, Skov RL, Holmes M (2012). Development of a real-time quadruplex PCR assay for simultaneous detection of nuc, panton-valentine leucocidin (PVL), mecA and homologue mecALGA251. J Antimicrob Chemother.

[26] R Core Team. R: A Language and Environment for Statistical Computing. https://www.R-project.org/. Accessed 16 Mar 2023.

[27] Kobusch I, Müller H, Mellmann A, Köck R, Boelhauve M (2020). Single blinded study on the feasibility of decontaminating LA-MRSA in pig compartments under routine conditions. Antibiotics.

[28] Elstrøm P, Grøntvedt CA, Gabrielsen C, Stegger M, Angen Ø, Åmdal S (2019). Livestock-associated MRSA CC1 in norway; introduction to pig farms, zoonotic transmission, and eradication. Front Microbiol.

[29] Parrott LJ (1992). Water absorption in cover concrete. Mater Struct.

[30] International Organization for Standards. ISO 4833–2:2013. Microbiology of the food chain—horizontal method for the enumeration of microorganisms—part 2: colony count at 30 °C by the surface plating technique. 2013.

[31] Quinn PJ, Markey BK, Leonard FC, FitzPatrick ES, Fanning S, Hartigan PJ (2011). Veterinary microbiology and microbial disease.

[32] Frank JF, Koffi RA (1990). Surface-adherent growth of *Listeria monocytogenes* is associated with increased resistance to surfactant sanitizers and heat. J Food Prot.

[33] Lee JS, Bae YM, Lee SY, Lee SY (2015). Biofilm formation of *Staphylococcus aureus* on various surfaces and their resistance to chlorine sanitizer. J Food Sci.

